# A Novel Improved Thromboembolism-Based Rat Stroke Model That Meets the Latest Standards in Preclinical Studies

**DOI:** 10.3390/brainsci12121671

**Published:** 2022-12-05

**Authors:** Katarzyna Pawletko, Halina Jędrzejowska-Szypułka, Katarzyna Bogus, Alessia Pascale, Foroogh Fahmideh, Nicoletta Marchesi, Aniela Grajoszek, Edyta Olakowska, Jarosław Jerzy Barski

**Affiliations:** 1 Department of Physiology, Faculty of Medical Sciences in Katowice, Medical University of Silesia, Medyków 18, 40-752 Katowice, Poland; 2 Department for Experimental Medicine, Medical University of Silesia, Medyków 4, 40-752 Katowice, Poland; 3 Department of Histology, Faculty of Medical Sciences in Katowice, Medical University of Silesia, Medyków 18, 40-752 Katowice, Poland; 4 Department of Drug Sciences, Pharmacology Section, University of Pavia, Viale Taramelli 14, 27100 Pavia, Italy

**Keywords:** focal ischemic stroke, photothrombosis, animal model, motor test, penumbra

## Abstract

The animal thromboembolic model of ischemia perfectly mimics human ischemic stroke which remains the leading cause of disability and mortality in humans. The development of new treatment strategies was therefore imperative. The purpose of this study is to improve the thromboembolic stroke model in rats in order to design experiments that use motor tests, and are in accordance with the 3R principles to prevent complications and maintain the same size of the infarct repeatedly. Tail vein dye application, a protective skull mask and a stress minimization protocol were used as additional modifications to the animal stroke model. These modifications significantly minimized the pain and stress severity of the procedures in this model. In our experimental group of Long-Evans rats, a photo-induced stroke was caused by the application of a photosensitive dye (Rose Bengal) activated with white-light irradiation, thus eliminating the need to perform a craniotomy. The animals’ neurological status was evaluated using a runway elevated test. Histological examination of the brain tissue was performed at 12, 24 and 48 h, and seven days post-stroke. Tissue examination revealed necrotic foci in the cortex and the subcortical regions of the ipsilateral hemisphere in all experimental groups. Changes in the area, width and depth of the necrotic focus were observed over time. All the experimental groups showed motor disturbances after stroke survival. In the proposed model, photochemically-induced stroke caused long-term motor deficits, showed high reproducibility and low mortality rates. Consequently, the animals could participate in motor tests which are particularly suitable for assessing the efficacy of neuro-regenerative therapies, while remaining in line with the latest trends in animal experimental design.

## 1. Introduction

Cerebral ischemic disease, still a common form of stroke, remains one of the leading causes of morbidity and mortality in humans [1]. Worldwide, 87% of all strokes are ischemic. These strokes are the second leading cause of death and the third leading cause of disability in the world [2]. In 2019, ischemic stroke accounted for 62.4% of all new strokes and 50% of its survivors are chronically disabled [1,3,4]. The only effective method of treating ischemic stroke is to quickly restore circulation in the hypoxic area (the penumbra) using modern methods such as thrombolysis or thrombectomy. Unfortunately, the “time window” for this type of therapy is short. Exceeding that window makes it impossible to use this type of treatment [5]. If perfusion is not re-established in time, the penumbra will gradually undergo infarction. This event is known as the “expansion of the core”, where tissue damage is deepened and worsened [6]. The strive to save the penumbra needs to be balanced against the risks of treatment. Thrombolytic drugs can indeed exacerbate the impairment of the blood–brain barrier that occurs with ischemia. Unfortunately, some thrombolytic agents have shown some undesirable side effects [7]. The penumbra is an area to be saved if possible, using methods such as thrombolysis and thrombectomy, when the “time window” is not exceeded. The methods to stretch that “time window” are the basis for many research ideas that seek after new neuroprotective treatments. The main purpose of this research is to set up a procedure able to save the penumbra and reduce the negative consequences of ischemic stroke such as disability [8]. Focal cerebral ischemia (FCI) is the closure of small vessels in the brain leading to microinfarcts. Blood flow is reduced in a very distinct specific region of the brain [9]. These strokes do not cause death but subclinically impair cognition, including memory impairment, and induce behavioral and motor changes [10]. 

In 1985, the photothrombotic model was introduced by Watson et al. to make a more reproducible cortical infarct without performing craniotomy in rats. The essence of this model is to use a photosensitive dye, for instance Bengal Rose (BR). The dye must be put into the bloodstream prior to irradiation. Illumination activates the dye, which leads to endothelial damage followed by platelet adhesion and aggregation. As a consequence, blood clots form in small vessels. The light passes freely through the bones of the skull, therefore craniotomy is not necessary [11]. An indisputable advantage of this model is the high similarity to the thrombotic process occurring in clinical conditions [11,12,13]. Generally speaking, an ideal animal model of FCI should be simple to perform, minimally invasive, repeatable, free from complications and applicable to more than one animal species. The model should also be low-cost and require an acceptable workload [13]. Moreover, a significant problem in the objective assessment of ischemia is the continuous and dynamic nature of changes in the size of the area of mild ischemia, the penumbra zone and the area of necrosis [14]. Therefore, improving the reproducibility of the location, shape and volume of the infarct area is very important for future preclinical studies. Thus, the development of an animal model that perfectly mimics FCI in humans will allow for the performance of reliable preclinical studies and the development of effective therapy for patients who cannot undergo thrombolysis or thrombectomy. 

The aim of our research was to modify the current photothrombotic animal model of FCI in the rat. In our study, we focused on reducing the severity of procedures such as the route of dye administration, limiting the irradiation area and reducing stress during the entire experiment. The next goal of this study was to test our modifications that significantly improve the quality of a multi-step analysis of the newly investigated neuro-protective therapies by using biochemical, histological, behavioral and motor analyses. 

## 2. Materials and Methods

### 2.1. Animals

The experimental protocols were approved by the Local Bioethics Committee at the Medical University of Silesia, Katowice, Poland, and conformed to international guidelines on the ethical use of animals. All rats were from our own breeding at the Department for Experimental Medicine, the Medical University of Silesia in Katowice, Poland. Thirty adult male Long–Evans (LE) rats weighing 220–240g were used. All animals were randomly divided into groups, namely: four experimental groups (*n* = 24, weight 226 ± 6 g) and two control groups: the SHAM (*n* = 3, weight 230 ± 10 g) and the Control Group (*n* = 3, weight 229 ± 9 g). In this experiment, four experimental groups were designed corresponding to the individual time interval–from the induction of ischemic stroke to the moment of euthanasia. Group A (*n* = 6) was placed at 12 h between the induction of ischemic stroke and euthanasia. Group B (*n* = 6) was placed at 24 h between the induction of ischemic stroke and euthanasia. Group C (*n* = 6) was placed at 48 h between the induction of ischemic stroke and euthanasia. Group D (*n* = 6) was placed at seven days between the induction of ischemic stroke and euthanasia. The two control groups used in this study were the SHAM group (*n* = 3), whose animals were subjected to the same procedure as the experimental ones but, instead of administering BR solution, they were given physiological saline solution, and the control group (*n* = 3) which consisted of the animals that did not undergo any procedure. The purpose of designing two control groups was to verify if the stroke procedure itself fails to cause side effects. Based on the latest trends, all experimental groups in this study (Groups A, B, C and D) were compared to one control group [15]. In order to verify some motor deficits, the animals from all groups underwent the horizontal runway elevated (HRE) test before the induction of ischemic stroke, after stroke and before euthanasia. After euthanasia, their tissue was collected for further histological determinations.

During the entire study, the animals were kept in a temperature-controlled and humidity-controlled room with a 12 h/12 h light/dark cycle and *ad libitum* access to water and standard rat chow. Chewing toys enriched the habitat of the animals. From the fifth week of the animals’ life until the start of the study, routine behaviors were implemented according to our protocol (see Supplementary Materials), which resulted in a significant stress reduction in the animals.

### 2.2. Animal Model

**Figure 1:**
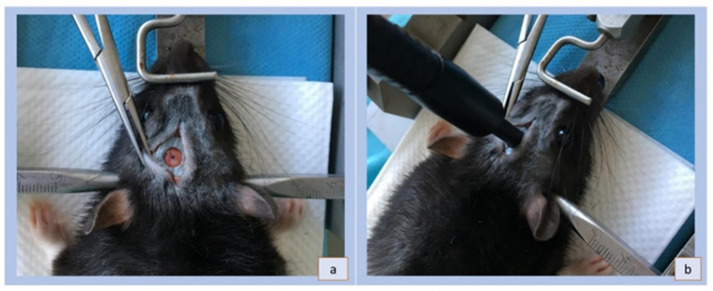
The photographs show the non-transparent mask used to protect the skull from light overexposure. The mask was made of a flexible 120 mesh artificial silk fabric covered with a layer of moisture and damage-resistant polyethylene. The fabric was totally opaque to block the light; (**a**) the mask fitted closely to the skull, revealing only an area intended for exposure; (**b**) the matching of the optical fiber with the mask before irradiation.

The animals during stroke induction surgery were put under general anesthesia induced by intraperitoneal administration of ketamine hydrochloride (100 mg/kg of body weight) and xylazine hydrochloride (10 mg/kg of body weight). The depth of the anesthetic effect was examined for the presence or absence of the corneal reflex, mustache reflex and foot-pinching reflex. Body temperature was maintained throughout the procedure at 37.5 °C by means of a heating mat. In this study, the localization of the infarct was produced in the posterior motor cortex. The location of the infarction, selected on the basis of our experience, coincides with previous publications [11,12]. During the procedure of stroke induction, the animals were placed on a heating mat; that allowed them to maintain a constant body temperature. After the rats were anesthetized and shaved, they were placed in a stereotaxic apparatus. The scalp was incised longitudinally with one incision (1.0–1.5 cm) to expose the skull, then the periosteum was removed and the skull bones were thoroughly cleaned to show sagittal sutures. The spot where the photo-induced impact was made was marked with the coordinates 0.5 mm in front of the bregma and 3.0 mm laterally from the center line according to the atlas by G. Paxinos and C. Watson (1986) [16]. The center was determined with optical fiber 5 mm in diameter. The area around the optical fiber was lined with a non-transparent mask (Figure 1) that protected the remaining areas from laser light. The diameter of the mask aperture coincides with the diameter of the optical fiber. Irradiation of the skull was carried out with white light with the following parameters: 560 nm and 3200 K [11] (KL2500 LCD SCHOTT, Mainz, Germany). The irradiation time was 15 min, and dye injection took place during the first minute of irradiation. The BR solution, 20 mg of BR in 1 mL of PBS (Sigma-Aldrich, St. Louis, MO, USA) at a dose of 1 mL/kg of body weight for each rat, was injected slowly through a pre-inserted polyethylene catheter into the tail vein (lateral caudal vein). After the entire dose of the solution was administered, the same amount of saline was administered. After irradiation was finished, the optical fiber and the catheter were removed and the incisions were sutured with skin sutures. After all surgical procedures, the animals were returned to their cages in a temperature-controlled room. Water and food were served *ad libitum* from the moment the animals were fully awakened.

### 2.3. Horizontal Runway Elevated Test

In this study, the motor deficits resulting from the induction of ischemic stroke in rats were tested by the HRE test. The HRE test is frequently used in animal models to evaluate forelimb and hindlimb stepping and coordination [16,17,18]. In this study the HRE test was performed in all groups except Group A (the time interval from general anesthesia to euthanasia was too short to perform a motor test correctly).

The HRE test involves the use of an elevated ladder apparatus and a camera (GoPro Hero 8, San Mateo, CA, USA). The camera is necessary to register the passage over the beam. These movies are needed for quantitative and qualitative analysis of rat gait. The HRE test in this experiment was constructed by us. The structure includes one horizontal metal slat (100 cm long × 4 cm wide) with 16-plane metal rungs and two cages (A-cage and B-cage). The rungs were placed on the slat at regular intervals of approximately 6 cm. The cages (A-cage and B-cage) were firmly attached to both ends of the metal slat. This connection allowed the rats to enter the cage directly from the slat after finishing their run. For each rat, cages A and B were provided with fresh substrate. To minimize the urge to jump off the slat, the entire HRE test foundation was raised to a height of 40 cm above the tabletop. The correct passage of the HRE test consisted of six runs for each animal: three runs from A-cage to B-cage and three runs from B-cage to A-cage. Rats started crossing the slat from A-cage to B-cage. This path was repeated in the opposite direction until the sixth correct pass was complete. The last run ended in A-cage for all rats. The performance of the HRE test was divided into two phases: the training phase (four days long; D1–D4) and the official passage phase (two days long; D5–D6). After the training, during the official passage phase, all rats performed one official pre-stroke passage on the day before stroke induction (D5) and one official post-stroke passage on the day of euthanasia (D6).

### 2.4. Sample Preparation and Nissl Staining

Histological evaluation requires the collection of brain tissue from rats. All samples were collected for each time group in accordance with the assigned time interval between the ischemic stroke induced and euthanasia. For Group A (*n* = 5), it was 12 h,; for Group B (*n* = 5), it was 24 h,; for Group C (*n* = 5), it was 48 h,; and for Group D (*n* = 5), it was seven days. In the SHAM Group (*n* = 3), the samples were collected 24 h after a SHAM ischemic stroke induction. The samples were also collected for the Control Group (*n* = 3). The rats were anesthetized and transcardially perfused with 4% paraformaldehyde. The fixed brains were removed and incubated at the same time overnight at 4 °C in a paraformaldehyde solution. Then, the rats’ brains were dehydrated, embedded in paraffin and finally sectioned on the microtome (Leica Microsystems, Mannheim, Germany) in coronal planes (−2.50 mm to −2.90 mm from bregma) in 7 µm-thick slices. The distance between 10 sections used per animal was 50 µm. Nissl staining was performed to locate the area of necrosis and the area of penumbra after ischemic stroke in the ipsilateral hemisphere. This type of staining made it possible to easily locate the ischemic area which is pale in staining compared to healthy tissues. Before the dye was administered, slide-mounted sections were taken at 0.5-mm intervals. Preparations were stained with 1 g/L cresyl violet dye (Sigma-Aldrich) in water, according to the classical protocol of Nissl staining with our own modification [19]. Images were acquired from the peri-infarct cortex area in the ipsilateral hemisphere using a digital camera (Olympus OM-D E-M10 Mark IV, Tokyo, Japan) together with a calibration slide for scale. The area of the infarct in each section, as identified by pale staining, was measured using ImageJ [20]. The infarct volume, width and depth for each brain were measured.

### 2.5. Statistical Analysis

The statistical analysis was performed using the Statistica 13.1 software (Dell, Austin, TX, USA). The significance level was assumed to be α = 0.05. The results are presented in accordance with the English notation, where the decimal separator is a point, in accordance with the APA standard. Descriptive statistics of the studied variables in groups were presented, tests examining the normality of distribution were carried out where appropriate, also Levene’s test for homogeneity of variance was performed. Based on these results, parametric (ANOVA, Student-*t*) or non-parametric (Kruskal–Wallis, Mann–Whitney and Wilcoxon’s) tests were selected and, when statistically significant results were obtained, appropriate post hoc tests (Bonferroni and Dunn) were carried out. The values are presented in charts as the means + standard deviations for variables analyzed with parametric tests and as medians along the quartile range (IQR) where the variables were analyzed with non-parametric tests.

## 3. Results

In all experimental groups, two rats died immediately after anesthesia administration, which gives 5.5% mortality in the entire study. Apart from that, no complications were found, even at the sites of inflammation and necrosis within the skin incision and at dye injection sites. The Nissl staining method allowed us to efficiently determine infarct volumes in the stained brain sections and to illustrate the size and location of photo-chemically induced ischemic infarcts.

### 3.1. Characterization of The Peri-Infarct Area following Ischemic Stroke

**Figure 2:**
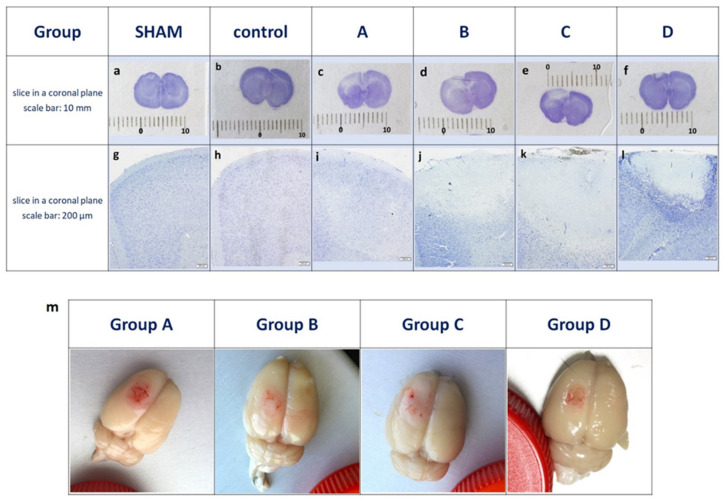
Frontal slices of rat brains following Nissl staining after photothrombotic ischemic stroke. (**c**–**f**) Pale-stained ischemic lesions in the left hemisphere. The brain tissue damage is visible in Groups A, B, C and D only in the ipsilateral hemisphere where focal ischemia was induced. No damage was reported in the contralateral hemisphere in Groups A, B, C and D. Scale bar: 10 mm; (**i**–**l**) Ischemic lesions in the area of the left hemisphere cortex in Groups A, B, C and DNissl staining. Scale bar: 200 µm; (**a**,**b**) In the SHAM and Control Group no areas of damage are visible in the ipsilateral and contralateral cerebral hemispheres; (**g**,**h**) Ischemic lesions in the area of the left hemisphere cortex in the SHAM and Control Group. Nissl staining. Scale bar: 200 µm; (**m**) The above view of the rat brains immediately after euthanasia following ischemic stroke in Groups A, B, C and D. Visible tissue damage in the left hemisphere.

Ischemic stroke was induced in all animals after the addition of BR and irradiation with white light. The use of a protective mask on all the animals shielded the specific area from irradiation and modulated a reproducible shape of the impact focus. Clear demarcation of the infarcted area was visible after 24 h post-stroke (Figure 2c–f). Histological examination revealed the areas of brain tissue damage in the experimental groups only in the ipsilateral hemisphere, while no damage was observed in the contralateral hemisphere (Figure 2c–f). Microscopic examination of the Nissl-stained tissues showed typical stroke-induced liquefactive necrosis on Day 1, infiltration of cells into the surrounding area of ischemia and detachment of the necrosis area by Day 7 (Figure 2i–l). Based on the volume of tissue exhibiting pale Nissl staining, after 12 h we observed that ischemic cells presented aberrant morphology; the boundary of the penumbra was still strongly blurred (Figure 2m).

**Figure 3:**
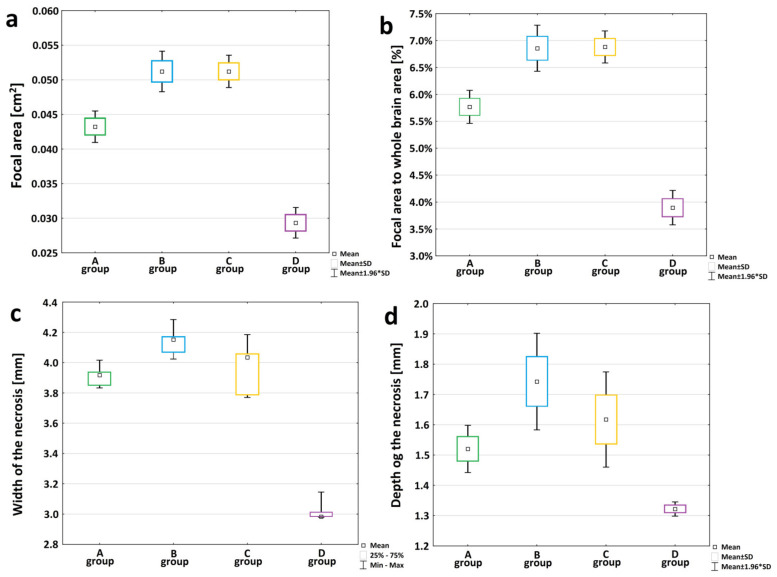
Comparison of ischemic area dynamics related to the changes after ischemic stroke induction in rats. Nissl staining; (**a**) the graph shows a comparison of the mean focal area [cm^2^] of necrosis within Groups A, B, C and D: ANOVA, F (3, 16) = 336.64, *p* = 0.000001, *post-hoc* Bonferroni’s test for each pair *p* < 0.001; (**b**) the graph shows a comparison between the focal area vs. the whole brain area [%] in Groups A, B, C and C: ANOVA, F (3, 16) = 322.59, *p* = 0.000001, *post-hoc* Bonferroni’s test for each pair *p* < 0.001; (**c**) the graph shows that the “width” of the necrosis spread after stroke induction differs among all pairs: Kruskal–Wallis test [H (3, *N* = 20) = 14.15429 *p* = 0.0027; Dunn’s *post-hoc* test indicates that there was a statistically significant difference between Groups B and D (*p* = 0.0014); (**d**) the graph shows that the “depth” of the necrosis spread after stroke induction differs among all pairs (following *post-hoc* Bonferroni’s test: for pair Group A vs. Group B *p* = 0.000171, for pair Group A vs. Group D *p* = 0.000578, for pair Group B vs. Group C *p* = 0.029973, for pair Group B vs. Group C *p* = 0.0000001, for pair Group C vs. Group D *p* = 0.000006), with the exception of Group A vs. Group C: no statistically significant difference following *post-hoc* Bonferroni’s test *p* = 0.135851.

In order to compare the size of the ischemic stroke area among different animal groups, the following parameters were measured: area of the entire section, area of necrosis, focal width and focal depth. For every rat, three measurements for each parameter were made and the average was calculated. These parameters have a normal distribution in the studied groups (Shapiro–Wilk test *p* > 0.07). Levene’s test indicates heterogeneity of variance within the groups for the variable “brain area” [F (3, 16) = 4.7379, *p* = 0.015]. Insomuch as the parameter “brain area” (area of the brain cross-section/(cm2)), an alternative non-parametric Kruskal–Wallis test was performed, showing no statistically significant differences among all the study groups in terms of the size of brain surface area (section): H (3, *N* = 20) = 3.5367, *p* = 0.3160. That means that the mean area of the sections at the tested time points did not change. The table presents the statistics of variables brain area [cm^2^] and the focal necrosis areas [cm^2^] of the examined rats in A, B, C and D groups available in Supplementary Materials. One-way ANOVA was applied for the other two variables: the “necrosis area” [cm^2^] (Figure 3a) and the “focal area to whole brain area” [%] (Figure 3b). The results show statistically significant differences among the study groups. Specifically, Group A had statistically significantly smaller areas of necrosis than Groups B and C (even greater than those occurring in Group D after the procedure). Moreover, the areas of necrosis in Groups B and C did not differ, although they were significantly greater than those observed in Group D (Figure 3a). Similar differences were found in the percentage of the necrotic area in relation to the area of the whole brain section (Figure 3b). The focal width and focal depth variables were characterized by normal distribution in the study groups (*p* > 0.17 in the Shapiro–Wilk test). An exception was the measurement of the necrosis length in Group D (*p* = 0.006). The “width” variable did not have a homogeneous variance in the studied groups when assessed with the Kruskal–Wallis test (Figure 3c). However, following Dunn’s post hoc test, the “width” variable shows a statistically significant difference between Groups B and D (*p* = 0.0014). The variable describing the focal “depth” was tested using a one-way analysis of variance which shows a statistically significant difference in the mean depth to which necrosis spread (F (3, 16) = 42,526, *p* = 0.00000). The post hoc Bonferroni’s test indicates that the “depth” to which necrosis spread after the induction of ischemic stroke differs among all pairs (each group is different), with the exception of Group A and Group C, where no statistically significant differences were observed (Figure 3d). For both parameters, the width and depth of necrosis, necrosis increased over time, reaching its peak at 24 h post-stroke (in Group B) and then dropped to or below the level observed at 12 h (in Group A). Concerning the control groups, a histologic examination revealed no areas of brain tissue damage in SHAM-operated rats in the ipsilateral or contralateral hemispheres (Figure 2a,b,g,h). Basic statistics for the measurements of the circumference of the whole brain in the SHAM and Control Groups were calculated and the results indicated that the brain surface areas were similar in both groups. The detailed table presents the statistics of variables “brain area” expressed in cm^2^ of the examined rats in the SHAM and Control Groups available in Supplementary Materials.

### 3.2. Characteristics of Motor Changes following Ischemic Stroke

The HRE test was performed to compare the effect of an enlarged area of necrosis in the motor cortex on the level of motor performance. All post-ischemic stroke animals performed a motor test; none of them exhibited any health problems, making it possible to perform the test; none of them were excluded from the test either. In order to analyze the progression of changes in the mobility measured by the HRE test, Groups B, C and D were compared in terms of both the number of paw slips/errors (each paw separately) and the average time to pass over the slat. The comparison was calculated for the results obtained between the day before the induction of ischemic stroke (D5) and the day after the induction(D6). Before ischemic stroke induction, after four days of training (D1–D4), the rats traversed the slat without error. After ischemic stroke, deteriorated mobility was observed in some rats.

**Figure 4:**
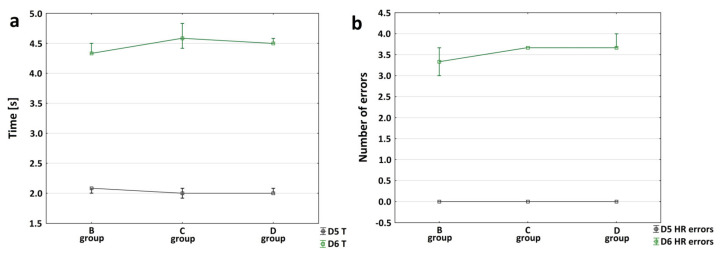
Comparison of the effects of an enlarged area of necrosis in the motor cortex on the level of mobility in rats after ischemic stroke; (**a**) the graph shows the median +/− IQR of the mean time (*t*) taken by the different groups of rats (B, C and D) to pass over the slat on test Day 5 (D5) and Day 6 (D6); the Wilcoxon test shows that for each group (the time to cross the beam is statistically different between D5 and D6 (*p* = 0.0431); (**b**) the graph shows the median +/− IQR of the average number of right hindlimb errors (HR-errors) made by the rats while crossing the slat. Comparison of the number of errors between Day 5 (D5) and Day 6 (D6) within all time groups (Groups B, C and D): the Wilcoxon test shows that on Day (D6), for Groups B, C and D, the number of HR-errors was statistically significantly greater than on Day (D5) (*p* = 0.0431).

To assess the variability between the measurements at D5 and D6, the Wilcoxon test was performed (a non-parametric alternative to the Student’s *t*-test for dependent variables, chosen due to zero-case values on D5; there were no errors during the test or no non-differing values). Normal distribution was not expected, since the number of trips on D5 was 0 for all. Then the variability of the scores between the time groups was analyzed. These were not considered to be dependent measurements because they were derived from other animals (each animal, regardless of the time group, participated in only one correct passage consisting of six runs in the HRE test). The results of the Wilcoxon test for each time point are shown (referring to the number of HRE errors) in Figure 4a.

To analyze the progression of changes in mobility measured in the slat test, Groups B, C and D were compared in terms of the number of foot errors (each paws separately) and the mean time of passage over the slat on the day after ischemic stroke induction (D6). The normality of distribution of variables was tested with the Shapiro–Wilk test. Statistics were calculated only for non-zero observations; no errors were noticed for the left forelimb or hindlimb. Therefore, it was decided that a non-parametric test should be carried out, i.e., the Kruskal–Wallis test. The animals showed variation in the number of trips at different time points only concerning the right forelimb (*p* = 0.0215). Then Dunn’s post hoc test was performed for this variable, showing no statistically significant differences between the specific group pairs(for pair Group B vs. C *p* = 1.000000, for pair Group B vs. D *p* = 0.085132, for pair Group C vs. D *p* = 0.249601), as reported in Figure 4b.

The HRE test was also performed in the SHAM and Control Group. The obtained results were used for the validation process between the SHAM and Control Group. Specifically, the SHAM and Control Group were compared in terms of the number of paw errors and the average time taken to pass over the slat. The analysis, applying the Friedman test, of the changes over the time taken to pass the slat on consecutive days of the experiment shows a shortening in the time needed to cross the slat. The results obtained following the Friedman test were applied for the Control Group: ANOVA χ^2^ (*N* = 3, df = 5) = 13,932, *p* = 0.01605, and for the SHAM Group: ANOVA χ^2^ (*N* = 3, df = 5) =13,431, *p* = 0.01966. The Mann–Whitney U test shows no statistically significant differences in the mean time taken by the rats to pass the slat between the control and SHAM groups on each day of the experiment (*p* > 0.16).

**Figure 5:**
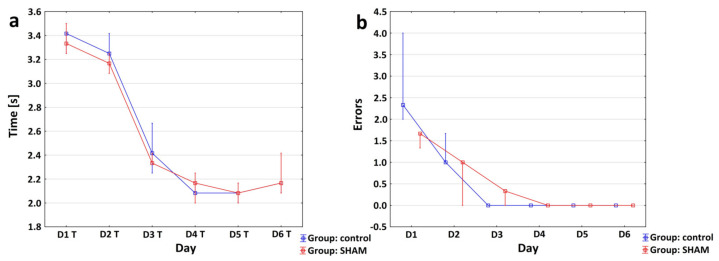
The validation process between the SHAM and Control Group with the use of the motor test; (**a**) the graph shows the median +/− IQR of the average time taken by control and SHAM rats to cross theslat. The Mann–Whitney *U* test shows no statistically significant differences in the mean time of rats’ crossing the slat between the Control Group and the SHAM Group on each of the days of the experiment (*p* > 0.16); (**b**) the graph shows the median +/− IQR of the number of errors (collectively for four paws) made by the rats on consecutive days of the training (D1–D5) and on D6 in the SHAM and Control Group. Friedman ANOVA for the Control Group: χ^2^ (*N* = 3, df = 5) = 15.00000, *p* = 0.01036; for the SHAM group: χ^2^ (*N* = 3, df = 5) = 12.92208, *p* = 0.02412. The Mann–Whitney *U* test shows no statistically significant differences in the mean number of errors made by rats between the Control and SHAM Group on each of the days of the experiment (*p* ≥ 0.1).

For the SHAM and Control Group, the errors made by all paws were added together to better represent the number of errors (Figure 5a). Friedman ANOVA, as applied for the Control Group [χ^2^ (*N* = 3, df = 5) =15.00000 *p* = 0.01036] and the SHAM Group [χ^2^ (*N* = 3, df = 5) =12.92208 *p* = 0.02412], showed a statistically significant difference between the average number of errors made on consecutive days of the experiment. The Friedman test gave us inconclusive results, so another test was performed for verification; the Mann–Whitney U test showed no statistically significant differences between the Control and SHAM Group in terms of the mean number of errors made by rats on each experimental day (*p* ≥ 0.1; Figure 5b). Separate graphs shows the median time taken by the rats to cross the beam in the consecutive days of the experiment in the Control Group and in the SHAM group in Supplementary Materials.

## 4. Discussion

For preclinical trials using animal models of ischemic stroke, it is essential to create a model that best mimics the actual condition and the features of such trauma in humans [21,22]. An ideal animal model of ischemic stroke should cause intravascular coagulation in an uninjured blood vessel, resulting in a necrotic area and the penumbra surrounding it [11,23,24]. The penumbra is an area of hypoxia, currently under our investigation, as we plan to develop new therapeutic methods to prevent the spread of necrotic areas [25]. The above-mentioned animal model of ischemic stroke should also enable us to accurately determine the functional status and the sensory and motor abilities of the animal involved [26]. The neurological scoring systems currently used were first applied in humans and later modified. The most common neurological scoring systems are not sensitive enough to reliably reflect the motor deficits resulting from ischemic stroke in animals [27]. We therefore decided to modify the existing model of photo-induced ischemic stroke in rats to allow the use of motor tests in the assessment of the animals’ condition and the dynamics of changes in their motor deficits after ischemic stroke. Therefore, we modified the method of dye administration (Bengal Rose), which—until now—was not only invasive but also prevented the use of behavioral tests. To date, the dye was usually administered into the femoral vein, which involved putting animals to sleep, incising their skin, introducing the Bengal Rose and suturing the wound. That prevented scientists from conducting motor and behavioral tests on these animals [12,25,27,28,29].

In photo-induced stroke models, problems can occur in relation to the abrupt nature of thrombotic lesion formation. In its basic version, our model based on photo-induced stroke allowed for the formation of an area of permanent ischemia, or necrosis, which was free from penumbra or contained a small band of penumbra [11] due to intensified necrosis formation and tissue edema [23,30,31]. Therefore, our goal was to modify the model to obtain a necrotic area fixed in shape (here, ring-shaped [32]) and the largest possible penumbra. Specifically, to obtain the latter and increase the reproducibility rate after inducing ischemia, we designed and created a light-fast mask. The mask allowed us to increase the reproducibility rate of the shape of the ischemic focus and obtain an area of penumbra in all animals. Using the mask eliminates craniotomy [28,30] (a highly invasive procedure).We found that our method of photosensitive dye administration, irradiation andstereotactic parameters can improve the results and increase our model’s usability [27]. Currently, two highly important methods are used in the treatment of stroke: thrombolysis–which involves the administration of a substance to dissolve the clots formed (such as alteplase); and thrombectomy–to remove such clots. Unfortunately, a model using BR—due to blood clotting in multiple small vessels within the irradiated area—cannot be used to study the lesions developing after thrombectomy. We do not know to what extent the model could be used to study the lesions occurring after thrombolysis [31]. The model seems ideal in searching for neuroprotective therapies, where popular techniques such as thrombectomy or thrombolysis are not feasible within the available therapeutic window.

Most studies on ischemic stroke involve Sprague–Dawley (SD) rats which demonstrate extreme variability in the experimental brain infarct volume. This may be due to the fact that the structure of the middle cerebral artery in SD rats highly varies from one rat to another [21,32]. Nevertheless, previous studies on the FCI model using Long–Evans (LE) rats do not show any deviation or variability related to this rat strain [33,34,35,36,37,38]. We therefore chose to induce the ischemic stroke specifically in LE rats. They are quite easy to tame, quick to learn and memorize, and they eagerly participate in various motor and behavioral tests. Our research confirms that LE rats can get used to a routine, learn easily and show no aggression.

After histological analysis, our data did not show any statistically significant differences in the volume, width or depth of the ischemic area in LE rats from each time group (see Section 3.1). These rats gain weight and become overweight rapidly, so they are ideal for ischemic stroke models with comorbidities such as obesity, hypertension and diabetes [21,23,39,40]. The reproducibility of results, ease of cooperation in motor tests and the individual characteristics of LE rats—such as the tendency to get fat—are favorable when designing new studies involving animal models of ischemic stroke.

In our study, we evaluated the time-sensitive changes in the volume, width and depth of the necrotic focus. The results show that 12 h after the stroke was photo-induced, the infarction has already advanced in its stage (Figure 2m). The volume of necrosis reached its maximum at 24 h and kept decreasing until Day 7 (Figure 3a,b). These results are therefore comparable to the ones previously published publications [25,28,30] After FCI, the volume of the ischemic area in humans falls within the range of 4–14% of the whole brain [10]. Following our modifications to the model, the mean volume of the ischemic area after 24 h was 9.2%. This result confirms that the FCI model in rats mimics the stroke focus volume observed in humans. Considering the width of the necrotic area, we concluded that there were statistically significant differences between the study groups (Figure 3c). The depth of the necrotic area also statistically significantly differed in all groups except the Group A and Group C pair, where the results did not confirm any significant differences in the depth of the necrotic area (Figure 3d). This may be due to the dynamics of the formation of stroke focus which has the largest volume of the ischemic area after 24 h. Then the volume keeps decreasing until Day 7. Most FCI studies have focused on the acute phase of ischemia, usually referring to the infarct volume and the penumbra after 24 h following the onset of ischemia [25,27,33,41,42]. However, with ischemic stroke models, the problem still relates to a narrow or a non-existent penumbra zone at time intervals following the first 24 h [12,23,24,31,43,44,45]. It is especially important to assess the ultimate infarct size at later time points in the preliminary tests of new modifications, as lesion growth can be delayed [46]. Therefore, we decided to select three acute-phase time groups: at 12 h (Group A), at 24 h (Group B), at 48 h (Group C), and a chronic-phase time group at seven days (Group D). These time groups allowed us to verify the efficacy of the modifications made to obtain the penumbra after inducing ischemia. On Day 7 post-stroke, Group D showed no penumbra around the necrotic area. The most prominent band of penumbra was visible in the 24-h-group (Group B). Similar results pertaining to the penumbra size in the course of FCI have been already described [30]. As a result of our modifications, we observed the penumbra zone in all animals from Group A, B and C. The results suggest that the animal FCI model is perfect for studies focused on the acute phase of ischemic stroke.

The severity of the sensory and motor deficits can be assessed using behavioral and motor tests, i.e., sticky tape removal test, cylinder test, forelimb placement test and beam balance test (beam walking assay) [47]. The performance of functional (behavioral, motor and cognitive) tests are important; even if the tissue is morphologically intact, the relative function may not be preserved and a delayed development of lesions cannot be excluded [48,49]. In our experimental approach, we chose a simple and easy horizontal runway elevated (HRE) test. Stroke focus in this experiment was located in the motor cortex, so with the HRE test we were able to monitor the motor deficits in rats surviving ischemic stroke. After the HRE test, we assessed the changes occurring after ischemic stroke by comparing the results in the period from Day 5 to Day 6 for each rat in terms of beam walking assay and the number of errors (i.e., paw slips). The results also allowed us to analyze the changes in the animals’ motor capabilities over time. On the fifth day of measurements (D5) none of the animals stumbled and all reached the shortest time to cross the beam. Day six (D6) was the day after stroke induction–at 24 h, 48 h and seven days, respectively. Moreover, at each time point (24 h, 48 h, seven days) only the number of right hind paw errors was significantly higher after stroke than before it (Figure 4b). The beam walking assay time was also statistically longer after stroke in all experimental groups than that on the day before stroke (Figure 4a). We observed over the days following ischemic stroke that the speed to cross the beam decreased and the level of foot incompetence, expressed by the number of errors, increased. All study groups showed motor dysfunctions after ischemic stroke induction and these persisted throughout the whole study. This is consistent with earlier studies [11,12,18,30,50]. However, the neurological scoring scale alone is not enough to accurately monitor the subtle changes in the animals’ motor abilities. Even a simple motor test is much more sensitive to show the motor changes than the most precise scoring scale. Precise stroke focus planning, in terms of its size and location, enables us to observe changes in the motor activity of a specific limb [29,51]. This allows for precise tracking of changes in motor deficits but also assessing progress in investigational neuroprotective therapies during each phase of ischemic stroke. In our study, the changes in limb mobility over consecutive days after ischemic stroke were determined based on the time of beam crossing and the number of foot errors made on D6, i.e., after stroke induction. Statistics were calculated for non-zero observations only. The results of the Kruskal–Wallis test showed that the animals exhibited variations in the number of stumbles at certain time points but only pertaining to the right front paw (*p* = 0.0215). In the next stage of analysis for this variable, a *post hoc* test (Dunn’s test) was performed. That test showed no statistically significant differences between group pairs. The results of the statistical analysis confirm the correlation between the animals’ mobility levels deteriorating over time after ischemic stroke. The HRE test was also performed for the SHAM Group and the Control Group. There were no statistically significant differences between these groups in the process of test acquisition, the number of foot errors or beam crossing errors (Figure 5b). The results confirm the validity of our previous assumptions regarding the minimization of the control group in this FCI model [15].

Directive 63/2010/EU states that education and training performed on live rats and mice are classified as animal experiments and require the implementation of the 3R principles [52,53]. These address replacement, reduction and refinement in animal testing [54,55]. The 3Rs have been extremely important for the development of good scientific standards and protection of animal welfare in search of reproducible reliable scientific results [54]. One of the 3Rs, “Replacement,” often requires a complex long-term strategy, while “Reduction” and “Improvement” can often be implemented immediately (in the short term) in individual experiments [53]. When modifying our photo-induced stroke model, we tried to make it compliant with the 3Rs. We put the strongest emphasis on reducing the number of animals involved and improving our method. The modifications were supposed to enhance the quality of the test performed by increasing its reproducibility, reducing invasive procedures and decreasing subject mortality. In particular, we focused on minimizing the invasiveness of the dye application and maximizing protection against unnecessary irradiation. The low mortality rate of only 5.5% demonstrated in our study, resulting from improved quality of the procedures, is in line with the Improvement principle [31]. In our study, the animals died only as a consequence of anesthesia administered before the surgery to induce ischemic stroke, not as a result of the experimental procedures. Based on the conducted experiments and the obtained results, we can draw the following conclusions about the SHAM Group: the whole procedure to apply the FCI model in rats, especially white-light irradiation of the skull, without the use of photosensitive dye, prevented lesions in the nervous tissue and other injuries to the adjacent tissue. This is also consistent with previous publications [18,30,55]. The absence of trauma in the motor cortex was confirmed by the results of motor tests. When comparing the Control Group with the SHAM Group, we observed no statistically significant differences in any HRE test results (Figure 5a,b). Therefore, when planning further studies using this experimental model, we suggest that only one control group should be designed, which will reduce the number of animals used in the study [30,31].

## 5. Conclusions

Photothrombotic stroke is a very well designed and unique model of occlusion in small cerebral vessels. The proposed refined method of modeling a local ischemic stroke has several advantages, as compared to the other models, i.e., ease of implementation, low mortality, highly reproducible lesion size and location, obtaining permanent structural and functional changes in the brain. Furthermore, it is free from complications and in compliance with the 3R principles. This model allows to perfectly monitor motor deficits whose changes can be observed within the time from the acute phase to the chronic phase. Of special interest is the monitoring of the chronic phase, which may be useful for the development of effective thrombolytic and neuroprotective therapies to counteract the damages associated with ischemic stroke.

## Data Availability

The dataset used and/or analyzed during this study is available from the corresponding author upon reasonable request.

## Supplementary Materials

The following supporting information can be downloaded at: https://www.mdpi.com/article/10.3390/brainsci12121671/s1; Table S1: Principles used to minimize stress in rats throughout the experiment; Table S2: The table presents the descriptive statistics of variables: BA is the brain area [cm^2^] and FA is the focal necrosis areas [cm^2^], FAtoBA is the focal necrosis area versus whole brain area [%] of the examined rats in Group A, B, C and D; Table S3: The table presents the descriptive statistics of the brain areas (BA) and focal necrosis areas (FA), expressed in cm^2^, in the examined rats in the SHAM and Control Group; Figure S1:The graph shows the median time taken by the rats to cross the slat on consecutive days of the experiment; (a) in the Control Group (Friedman test ANOVA χ^2^ (*N* = 3, df = 5) = 13.932, *p* = 0.01605); (b) in the SHAM Group (Friedman test ANOVA χ^2^ (*N* = 3, df = 5) = 13,431, *p* = 0.01966) [56,57,58,59,60].
