# Supplementary material for: A Novel Improved Thromboembolism-Based Rat Stroke Model That Meets the Latest Standards in Preclinical Studies
Source: Brain Sci. 2022 Dec 5;12(12):1671. doi: 10.3390/brainsci12121671 (PMC9776070; doi:10.3390/brainsci12121671)
Supplement: Supplementary file 1 [file brainsci-12-01671-s001.zip › brainsci-2062836-supplementary.pdf]

Supplemental materials:

Table S1. Principles used to minimize stress in rats throughout the experiment

Number of rule	Rules of conduct before and during the experiment with rats
1	*Habituation process :* In order to accustom the animals to the person performing the experiments and to the room where the tests will be performed, the habituation process was carried out from the end of the 5th week of age of the animals until the beginning of the test. All rats were habituated to the touch of the abdominal area by a human hand and exposing the abdomen. Overall, this procedure minimizes the stress associated with the intraperitoneal administration of anesthesia [1–4]. The entire procedure of habituation lasted the same time for all animals 7 weeks (from 5 weeks of age to 12 weeks of age of the rats).
2	*Award :* In order to relax the animals, a gentle tickling in the abdomen was used, which simulates playing between rats in a natural environment [5]. Tickling is a pleasant form of reward for rats, especially after performing motor and habit tests.
3	*Avoidance of new stimuli :* For habituation and activities, such as litter replacement or feeding, disposable cotton gloves were used. The smell and touch of rubber gloves caused discomfort in rats. In addition, cleaning and disinfection agents were odorless. To eliminate new and unknown odors, the person in contact with the animals did not use perfume, has not taken care of other animals, did not eat in working clothes.
4	*Routine :* The light phase and the dark phase, feeding, motor tests, control of breeding rooms and cleaners were always at the same time of the day.
5	*Familiar room :* Motor tests were performed in a room known and safe for the animals. This room belongs to a separate part of the breeding room. All rats were habituated therein to minimize stress.
6	*One person* : The same person who carried out the habituation process also performed the following activities: feeding, changing the litter, motor test, administering anesthesia, performing ischemic strokes, postoperative care. Minimizing the presence of other people allowed to avoid stress and distraction in the animals at any stage of the experiment.

Table S2. The table presents the descriptive statistics of variables: BA is the brain area [cm^2] and FA is the focal necrosis areas [cm^2] ,FAtoBA is the focal necrosis area *versus* whole brain area [%] of the examined rats in A, B, C and D groups.

Variable	Group	Valid N	Mean	Median	Minimum	Maximum	Lower Quartile	Upper Quartile	Std.Dev.
BA [cm^2]	D	5	0.753	0.750	0.744	0.763	0.748	0.761	0.008
FA [cm^2]	D	5	0.029	0.030	0.028	0.030	0.029	0.030	0.001
FAtoBA [%]	D	5	0.039	0.039	0.037	0.041	0.038	0.040	0.002
BA [cm^2]	C	5	0.744	0.747	0.735	0.750	0.740	0.748	0.006
FA [cm^2]	C	5	0.051	0.051	0.050	0.053	0.050	0.052	0.001
FAtoBA [%]	C	5	0.069	0.069	0.067	0.071	0.068	0.069	0.002
BA [cm^2]	B	5	0.747	0.748	0.740	0.750	0.746	0.750	0.004
FA [cm^2]	B	5	0.051	0.052	0.049	0.052	0.051	0.052	0.002
FAtoBA [%]	B	5	0.069	0.070	0.065	0.070	0.068	0.070	0.002
BA [cm^2]	A	5	0.749	0.750	0.746	0.753	0.748	0.750	0.003
FA [cm^2]	A	5	0.043	0.043	0.042	0.045	0.042	0.044	0.001
FAtoBA [%]	A	5	0.058	0.057	0.056	0.060	0.057	0.058	0.002

Table S3. The table presents the descriptive statistics of the brain areas (BA) and focal necrosis areas (FA) expressed in cm^2 of the examined rats in the SHAM and control groups.

Variable	Group	Valid N	Mean	Median	Minimum	Maximum	Lower quartile	Upper quartile	Std.Dev.
BA [cm^2]	control	3	0.752	0.751	0.750	0.755	0.750	0.755	0.0027
FA [cm^2]	control	3	0.000	0.000	0.000	0.000	0.000	0.000	0.0000
BA [cm^2]	sham	3	0.749	0.750	0.747	0.751	0.747	0.751	0.0020
FA [cm^2]	sham	3	0.000	0.000	0.000	0.000	0.000	0.000	0.0000

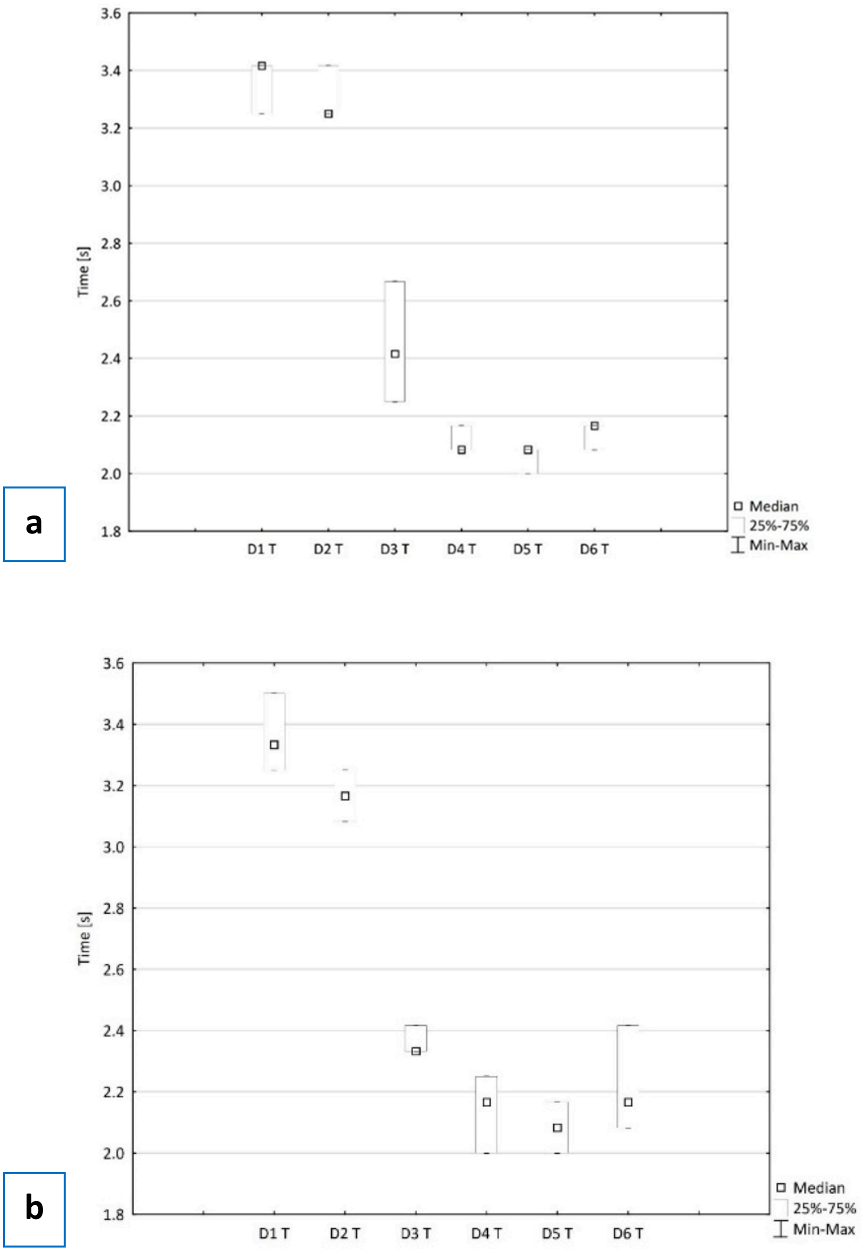

Figure S1. The graphs shows the median time taken by the rats to cross the slat in the consecutive days of the experiment; **(a)** in the control group [Friedman's test ANOVA $\chi 2$ (N = 3, df = 5) = 13.932, p = 0.01605]; **(b)** in the SHAM group [Friedman's test ANOVA $\chi 2$ (N = 3, df = 5) = 13,431, p = 0.01966].

Bibliography for supplemental materials:

1. Leussis, M.P.; Bolivar, V.J. Habituation in Rodents: A Review of Behavior, Neurobiology, and Genetics. *Neurosci. Biobehav. Rev.* 2006, *30*, 1045–1064.

2. Dissegna, A.; Turatto, M.; Chiandetti, C. Context-Specific Habituation: A Review. *Animals* 2021, *11*.

3. Parsons, M.H.; Sarno, R.J.; Deutsch, M.A. A Detailed Protocol to Enable Safe-Handling, Preemptive Detection, and Systematic Surveillance of Rat-Vectored Pathogens in the Urban Environment. *Front. Public Heal.* **2016**, *4*, doi:10.3389/fpubh.2016.00132.

4. Lydén, F. *Handling Methods of Laboratory Mice and Rats Hanteringsmetoder Av Försöksmöss Och Råttor Uppsala 2016 Ethology and Animal Welfare-Bachelor’s Programme*; Helmersson, 2016;

5. Cloutier, S.; LaFollette, M.R.; Gaskill, B.N.; Panksepp, J.; Newberry, R.C. Tickling, a Technique for Inducing Positive Affect When Handling Rats. *J. Vis. Exp.* **2018**, *2018*, 1–10, doi:10.3791/57190.
